# Cooperative Fusion Based Passive Multistatic Radar Detection

**DOI:** 10.3390/s21093209

**Published:** 2021-05-05

**Authors:** Asma Asif, Sithamparanathan Kandeepan

**Affiliations:** School of Engineering, RMIT University, Melbourne 3000, Australia; kandeepan.sithamparanathan@rmit.edu.au

**Keywords:** multistatic passive radars, singular value decomposition, fusion, detection, statistical testing

## Abstract

Passive multistatic radars have gained a lot of interest in recent years as they offer many benefits contrary to conventional radars. Here in this research, our aim is detection of target in a passive multistatic radar system. The system contains a single transmitter and multiple spatially distributed receivers comprised of both the surveillance and reference antennas. The system consists of two main parts: 1. Local receiver, and 2. Fusion center. Each local receiver detects the signal, processes it, and passes the information to the fusion center for final detection. To take the advantage of spatial diversity, we apply major fusion techniques consisting of hard fusion and soft fusion for the case of multistatic passive radars. Hard fusion techniques are analyzed for the case of different local radar detectors. In terms of soft fusion, a blind technique called equal gain soft fusion technique with random matrix theory-based local detector is analytically and theoretically analyzed under null hypothesis along with the calculation of detection threshold. Furthermore, six novel random matrix theory-based soft fusion techniques are proposed. All the techniques are blind in nature and hence do not require any knowledge of transmitted signal or channel information. Simulation results illustrate that proposed fusion techniques increase detection performance to a reasonable extent compared to other blind fusion techniques.

## 1. Introduction

Passive radars have been an emerging field for researchers since last many years. They have many additional perks in contrast to active radars; the noteworthy one is the reduced cost of implementation since they use transmitters like FM broadcasting signals, television transmission signals, or can be mobile phone signals. Therefore, no extra hardware for signal generation is needed [1,2]. Another perk of passive radar is non-traceability, as it uses the signal of opportunity. The shortcoming of passive radar is that there is no perfect knowledge of the transmitted signal. However, the noisy version of the transmitted signal is accessible through the reference channel.

A radar system with multiple receivers and multiple illuminators (IOs) is called a passive multistatic radar system. Multiple input Multiple output (MIMO) radars are passive multistatic radars, exploiting multiple IOs and multiple receivers, spatially separated to each other [3]. In general, a passive multistatic radar system’s performance is better than passive bi-static radars because the number of data samples received at spatially separated receivers is always more [4]. Hence, there is an increase in the detection capabilities of the system [5].

The IOs are non-cooperative in nature, making the transmitted signal unknown to the receiver. Therefore, matched filters that are commonly used for detection in radar systems are not a good choice. However, we have multiple receivers, spatially distributed, collecting echos of the target simultaneously in a passive multistatic radar system. The correlation that exists between these echos can be used for detecting the targets. To exploit this correlation, we have used a mathematical tool called Random Matrix theory (RMT). The theory mainly deals with different forms of random matrices and the properties of eigenvalues and eigenvectors obtained from the sample covariance matrices (SCM). In our research, we have used singular values to design six novel soft fusion techniques. RMT has been used, as it has evolved as an excellent tool that can be used in signal processing [6]. Moreover, detection problems are widely addressed in random matrix theory [7]. Furthermore, the detection methods derived from the random matrix theory are among the best detection methods in cognitive radios [8,9].

One of the hot topics of research in radar, sonar, and communication engineering like cognitive radios is detection [10,11]. Detectors are the hub of radar systems and have extensively been addressed in the past [12,13]. The most commonly used detection method is cross-correlation. However, there are several other methods in the research as well. Singular vector-based methods have gained quite a popularity in the recent past [14]. SSVD [15] is one of the recent contributions, where singular value decomposition (SVD) is used to decompose the received data matrices. The resulting singular vectors are formulated to form a detection test statistics, comprising all the singular vectors obtained from every channel in the system.

In communication theory, when spatially separated multiple receivers observe a common phenomenon, this is known as fusion. It has been widely used in cooperative spectrum sensing [10]. In fusion, a group of *P* nodes cooperates by sharing their own local sensing decisions on a target’s presence. The local sensor’s local decisions can be either soft or hard decisions. A global verdict on the target’s presence is then made by fusing the data received from all the local nodes. The central unit where the decision is made is known as the fusion center, and the fusion is done by using an appropriate fusion strategy.

Different types of transmission impairments are very common to occur during the process of spectrum sensing. This includes rigorous fading [16], issues that arise due to hidden terminals [17] and shadowing effect [18]. The impairments cause unfavorable effects on the sensing channel and bring out a drastic loss in the system’s detection performance. More precisely, the presence of shadowing and multi-path fading causes serious signal attenuation, making it hard for the detector to distinguish between the state of the target under a low signal-to-noise ratio (SNR). Furthermore, the variation of interference sources causes noise fluctuation, and the performance of energy detection decreases due to the deviation of estimated noise. Detection results become unreliable if the SNR of the received signal is less than a certain threshold [17]. As mentioned earlier, cooperative fusion is an assuring method to suppress most of the mentioned problems [19]. Nevertheless, it is notable that under the condition of a low signal-to-noise ratio, the fusion results are adversely affected and causes serious degradation of the detector’s performance [20]. Therefore, the channel between the transmitter and receiver should be considered a major effecting factor, and hence the corresponding weights in the fusion method need accurate quantization.

In this paper, we exploit spatial diversity by using multiple receivers in a multistatic passive radar system; each receiver acts as a local detector. The local decision is sent to the fusion center, where fusion techniques are applied, and a final decision about detection is made.

The main contributions of this paper are:

- A framework for data fusion in multistatic radar systems with hard and soft fusion methods.
- Proposal of novel soft fusion techniques based on random matrix theories for improving the detection performance.
- Theoretical treatment of a known soft-decision fusion method “the equal gain combining” method.
- Simulations of known hard-decision fusion and soft decision fusion techniques and comparing the detection performance.

## 2. Brief Review of Existing Work on Passive Multistatic Radar

The performance of the system is increased in the recent past by deploying multiple receivers. MIMO radar is an excellent example of multiple receivers. An intensive study on distributed MIMO radars, a sort of multistatic radar, has been presented in [21]. As compared to conventional radars, MIMO radars have the perks of better detection performance, increased coverage, and higher accuracy of target localization [21,22]. In [23], a detection algorithm based on the fusion of signal for frequency diversity MIMO radar is proposed. The proposed algorithm secured a unique and better solution for the given optimizations and improved detection performance at lesser communication expense. In [3,24],the passive multistatic radar is indicated as passive MIMO radar as it utilizes multiple illuminators as well as multiple receivers spatially separated from each other. The case of multiple illuminators in passive radars has been studied in [25,26] for localization of the target.

Passive multistatic radars can also be referred to a distributed systems where original signals received at the local detectors are sent to a fusion center for the final decision. In cognitive radio networks, fusion methods have been widely used for cooperative spectrum sensing to detect primary user [10]. For this purpose, several fusion schemes have been presented in the literature, which involves combining local sensing information of the secondary users [27]. Based on the combining technique, fusion can be categorized as hard and soft fusion. Due to energy and bandwidth efficiency and comparable performance with the soft schemes, in [28], hard decision fusion schemes have been chosen for performance up-gradation by selecting an optimal local detector. There are many hard fusion techniques proposed in the literature [29,30]. The OR and AND rule are most noticeable since they are easy to implement by simple logic. However, the OR and AND rule can be considered special cases of the general Q-out-of-P rule when Q is either 1 or P, respectively. For the case of sensors, in [31], the spectral efficiency of the wireless sensor network has been increased by applying channel aware decision fusion. In another study [32], the position of the target is estimated via joint use of time difference of arrival and passive coherent locator measurements, respectively.

Soft fusion has the potential to adapt, with a trade-off, i.e., increase data size being transmitted to the fusion center. Soft fusion-based techniques are commonly used in the cognitive radio environment. In [33], a novel collaborative sensing method is proposed to gather the local sensor’s results and the estimate of SNR and select the local sensor with high SNR for a final decision. In [34], the average SNR of each cognitive radio user at the receiver is adapted to determine the detection test; afterwards, the detection threshold is optimized to decrease the error probability. In [35], a weighted soft fusion algorithm is proposed, where the maximum weight is assigned to the node with the highest SNR. In another study [36], to deal with the interference of primary user and increase the throughput rate, an interference-aware model for CSS is introduced and investigated for the issue of sensing-throughput trade-off. Based on a careful study of fusion gain, nodes lasting power, distance from primary user, and the implementation expenses of sensing and reporting, a study [37] is proposed based on a shared dynamic load-balancing algorithm. Considering the variance and spatial location and topological formation between the cooperative sensors, a fusion method based on location reliability is proposed [38]. Most of the literature dealing with multiple receiving nodes assumes that the SNR between all the secondary users and the primary users are same and ideal.

In cognitive radios, the prevailing soft fusion method mainly weighs measured power received from secondary users and designs optimal weight coefficients for various channels. In [39], for slow fading channels, a revised linear fusion approach based on deflection coefficient was proposed. Ref. [40] introduces an optimal soft fusion technique assuming that the primary signal is Gaussian and SNR is known. Furthermore, it was concluded that the maximal ratio combining (MRC) and the equal gain combining (EGC) [41] soft fusion techniques are near-optimal at low and high signal to noise ratios, respectively. In [33], an optimal soft fusion technique is proposed assuming a deterministic but unknown primary signal. However, the algorithms proposed in the literature require the statistical attributes of the power received under both hypotheses.

Singular values decomposition (SVD) is a broadly used signal processing tool [42]. In detection problems of radar, like in [43], noise is removed from the received data using SVD. In another research, [14], a singular vector-based detector has been proposed by decomposing the received data matrices using SVD. The detector mainly exploits the low-rank waves traveling inside the transmitted waves and proves to perform better than the rest of the detectors.

The research work in this paper adopts the idea of fusion techniques and proposes new soft-fusion methods that outperform other methods in the literature.

## 3. System Modeling

Cooperative sensing for target detection in radar system is a two-level decision process to decide on the presence of a target in the environment. The first level of decision-making is at the receiver node known as the local detector whereas the second level of decision making takes place at the fusion center. The complete system model of local detector and fusion center has been explained in the following sub-sections.

### 3.1. Local Detector

**Figure 1:**
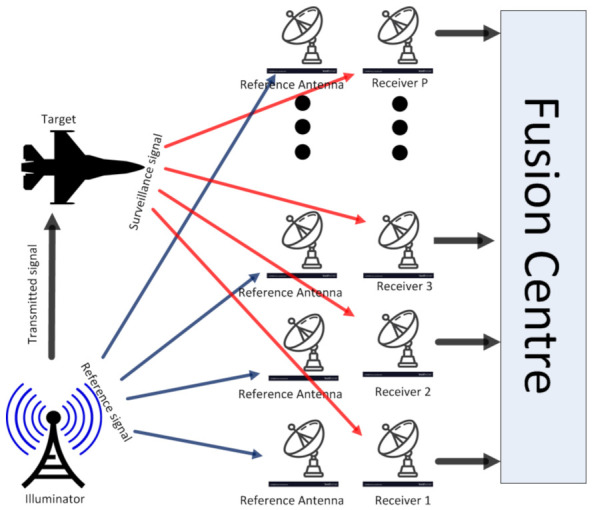
System model considered for passive multistatic radar.

Our system model mainly consists of a single static target and multiple receivers, as depicted in Figure 1. Here, we consider a single illuminator of opportunity (IO) as transmitter, and multiple pairs of receiving antennas, receiving through surveillance channel and reference channel, separately. We also assume that the signal we receive is Doppler and delay corrected. We take into account statistical testing methods based on hypothesis for detecting the presence of a target. Suppose *P* is the total number of local detectors present in the system, hence, the detection problem can be formulated as the following hypothesis test,

(1) $$H_{po}:\left\{\begin{array}{c} y_{s_{\left(p,i\right)}}=z_{s_{\left(p,i\right)}}\\ y_{r_{\left(p,i\right)}}=\mu_{r_{\left(p,i\right)}}u+z_{r_{\left(p,i\right)}} \end{array}\right.$$

(2) $$H_{p1}:\left\{\begin{array}{c} y_{s_{\left(p,i\right)}}=\mu_{s_{\left(p,i\right)}}u+z_{s_{\left(p,i\right)}}\\ y_{r_{\left(p,i\right)}}=\mu_{r_{\left(p,i\right)}}u+z_{r_{\left(p,i\right)}} \end{array}\right.$$

where i∈{1,⋯,N} symbolizes the number of snapshots. p∈{1,⋯,P} denotes the number of local detectors. Here, *s* and *r* describes the surveillance channels and reference channels, respectively. The terms $\mu_{s_{\left(p,i\right)}}$ and $\mu_{r_{\left(p,i\right)}}$ are attenuation coefficients, considered to be statistically independent and Gaussian distributed with zero mean and variance $\sigma_{s}^{2}$ and $\sigma_{r}^{2}$, respectively, [14]. Moreover, $z_{s_{\left(p,i\right)}}$ and $z_{r_{\left(p,i\right)}}$ is the Gaussian distributed noise in both the channels, respectively. The vector u is the transmitted waveform assumed to be of length *M* samples, normalized and unknown. It is noteworthy that distinguishing the pulses from one another for the formulated problem is only possible when there is perfect time synchronization between the transmitter and receiver. Commercial transmitters can achieve this kind of perfect synchronization by following certain mechanisms. However, in this paper, we have considered the case of perfect time synchronization.

Converting Equations (1) and (2) into matrix form by concatenating *N* time snapshots at the $p^{th}$ local detector, we can have our hypothesis in the form of,

(3) $$H_{po}:\left\{\begin{array}{c} Y_{s_{\left(p\right)}}=Z_{s_{\left(p\right)}}\\ Y_{r_{\left(p\right)}}=\mu_{r_{\left(p\right)}}U+Z_{r_{\left(p\right)}} \end{array}\right.$$

(4) $$H_{p1}:\left\{\begin{array}{c} Y_{s_{\left(p\right)}}=\mu_{s_{\left(p\right)}}U+Z_{s_{\left(p\right)}}\\ Y_{r_{\left(p\right)}}=\mu_{r_{\left(p\right)}}U+Z_{r_{\left(p\right)}} \end{array}\right.$$

where $Y_{s_{\left(p\right)}}=\left[y_{s_{\left(p,1\right)}},\cdots ,y_{s_{\left(p,N\right)}}\right]$ and $Y_{r_{\left(p\right)}}=\left[y_{r_{\left(p,1\right)}},\cdots ,y_{r_{\left(p,N\right)}}\right]$. Similarly, U=[u,⋯,u]. Furthermore, attenuation terms are concatenated to form $\mu_{s_{\left(p\right)}}$= diag$\left\{\mu_{s_{\left(p,1\right)}},\cdots ,\mu_{s_{\left(p,N\right)}}\right\}$, $\mu_{r_{\left(p\right)}}$= diag$\left\{\mu_{r_{\left(p,1\right)}},\cdots ,\mu_{r_{\left(p,N\right)}}\right\}$. The noise considered is independent and Gaussian distributed with zero mean and variance $\sigma^{2}$. Moreover, $\rho_{s}$ and $\rho_{r}$ serve as a signal to noise ratio present in both surveillance and reference channels, respectively, defined as,

(5) $$\rho_{s}=20\;\log\frac{\sigma_{s}}{\sqrt{M}\sigma }\;\mathrm{dB}$$

(6) $$\rho_{r}=20\;\log\frac{\sigma_{r}}{\sqrt{M}\sigma }\;\mathrm{dB}$$

#### 3.1.1. Hard Local Decision

The local decisions made by the local receiver nodes are hard decisions with a binary representation of −1 (for binary 0) for the case of deciding on $H_{0}$, i.e., the target is absent, and 1 for the case of deciding on $H_{1}$, i.e., the target is present. The techniques and strategies used for making such hard decisions are purely up to the receiver nodes. Suppose $\eta_{p}$ is the decision threshold at the $p^{th}$ receiver, then the corresponding hard decision $\omega_{i}\left(p\right)$ is given by,

(7) $$\omega_{i}\left(p\right):\left\{\begin{array}{c} \begin{array}{c} =1:T_{p}>\eta_{p}\\ =-1:T_{p}<\eta_{p} \end{array} \end{array}\right.$$

where $T_{p}$ is the test statistic used for the local receiver’s detection. Once the local receiver makes a decision, it then reports it to the fusion center.

#### 3.1.2. Soft Local Decision/Local Test Statistics

In soft fusion, each local detector will receive the signal and apply the local test statistics. When applying the fusion framework on multistatic radars, the local detector’s test statistic serves as a soft local decision. Therefore, in this section, we will present all the local detectors we have considered for analysis and comparison purposes. Suppose we receive information at the $p^{th}$ local detector, then $T_{p}$ is the corresponding local test statistics. Let us assume we have SVD as a local detector then,

(8) $$T_{p}={\left|v_{s_{\left(p\right)}}^{H}v_{r_{\left(p\right)}}\right|}^{2}$$

where $v_{s_{\left(p\right)}}$ and $v_{r_{\left(p\right)}}$ are the maximum left singular vectors of $Y_{s_{\left(p\right)}}$ and $Y_{r_{\left(p\right)}}$, respectively. The decomposition of $Y_{s_{\left(p\right)}}$ and $Y_{r_{\left(p\right)}}$ is given by,

(9) $$Y_{s_{\left(p\right)}}=V_{s_{\left(p\right)}}S_{s_{\left(p\right)}}U_{s_{\left(p\right)}}^{\prime }$$

(10) $$Y_{r_{\left(p\right)}}=V_{r_{\left(p\right)}}S_{r_{\left(p\right)}}U_{r_{\left(p\right)}}^{\prime }$$

here $V_{s_{\left(p\right)}}$ and $V_{r_{\left(p\right)}}$ represent the matrices containing left singular vectors, $S_{s_{\left(p\right)}}$ and $S_{r_{\left(p\right)}}$ represent the diagonal matrices containing singular values, $U_{s_{\left(p\right)}}$ and $U_{r_{\left(p\right)}}$ represent the matrices containing right singular vectors. These matrices are obtained by applying SVD on data received at both surveillance and reference antennas of $p^{th}$ detector, respectively.

To design a framework for data fusion in multistatic radars, we have analyzed the fusion techniques for the case of different local detectors already present in the literature. The details of the local detector considered are as follows.

Here, in this paper, SSVD [15], a recently proposed detector, has been investigated specifically for the case of fusion systems. SSVD is a RMT inspired detector formulated from singular vectors obtained from the received signal. The detector gives better detection probability and hence improves the system’s performance considerably compared to existing methods. The test statistics for the SSVD detector are given by,

(11) $$T_{SSVD}=\sum_{k=1}^{M}{\left|v_{sk}^{H}v_{rk}\right|}^{2}$$

here, $v_{sk}$ and $v_{rk}$ express left singular vectors calculated by decomposing the matrices $Y_{s_{\left(p\right)}}$ and $Y_{r_{\left(p\right)}}$ into their corresponding singular values and singular vectors. Here, k∈[1,⋯,M] as *M* is the highest rank that can be obtained. The degree of freedom (DF) of the SSVD detector is 2M and depends on the number of singular vectors used.

A singular vectors-based detector is expressed in [14]. The detector exploits the periodically repeating wave traveling on surveillance and reference channel in passive radars. Hence, singular values and singular vectors are obtained by decomposing the received data from both channels. The detector uses only the largest singular vector. SVD is also a particular case of SSVD detector where only the most significant singular vector is considered. The test statistic of the SVD detector is given by Equation (8).

The most commonly applied method for detecting a target in passive radar is cross-correlation, where we cross-correlate the data received from the surveillance and the reference channels, respectively. The mathematical expression for the detector is given by,

(12) $$\begin{array}{c} T_{CC}=\sum_{i=1}^{N}{\left|y_{si}^{H}y_{ri}\right|}^{2} \end{array}$$

where $y_{si}$ and $y_{ri}$ are the received vectors from both the surveillance and reference channels, respectively.

In this research, we consider a perfect case of the detector. It serves as a benchmark to compare the performance of other detectors. This detector is not a very practical case as it considers the transmitted signal to be known. The detector is called the clairvoyant detector. The test statistic of the clairvoyant detector is given by,

(13) $$\begin{array}{c} T_{Clair}=\sum_{i=1}^{N}{\left|y_{si}^{H}u\right|}^{2} \end{array}$$

where $y_{si}$ are the vectors we receive from the surveillance channel, and u is the transmitted signal.

The second decision process in target detection is performed at the fusion center after receiving all the local decisions from the local detectors. The final detection is done by fusing (depending on the reporting strategy) the received data and deciding upon the target’s presence. The mechanism of fusion done can be hard fusion or soft fusion. We categorize the fusing site into hard fusion-based detection or soft fusion-based detection depending on the first level’s strategy.

## 4. Hard Fusion Based Detection

Once we have local decisions made by the local detectors with a binary representation as explained in Section 3.1.1, we process this information in the hard fusion center. The most straightforward and commonly used fusion strategy for hard decision-based fusion is to pick on $H_{1}$ if at least Q out of the P number of local detectors has claimed $H_{1}$ to the fusion center. This strategy is known as Q-out-of-P rule.

Given that $\omega_{i}\left(p\right)$ are the reported hard decisions by the respective local detector, then the Q-out-of-P fusion rule is given by,

(14) $$d_{i}=\left\{\begin{array}{cc} \;\;1; & \;\;\;\;\;\;\;\;\nu \geq Q\\ -1; & \;\;\;\;\;\;\;\nu <0 \end{array}\right.$$

where ν is the test statistic at the fusion center given by,

(15) $$\nu =\sum_{p=1}^{P}0.5\left(\omega_{i}\left(p\right)+1\right)$$

The detection performance of the Q-out-of-P fusion technique can be easily analyzed by considering the detection and the false alarm probabilities of the local decisions from the local detector. Suppose if $P_{D}=P_{r}\left[\omega_{i}\left(p\right)=1|H_{1}\right],\forall \left(i,p\right)$ is the detection probability and $P_{FA}=P_{r}\left[\omega_{i}\left(p\right)=1|H_{0}\right],\forall \left(i,p\right)$ is the false alarm probability of the local decisions made at the local detector, then the overall miss-detection $P_{M,c}$ and the false alarm $P_{FA,c}$ probability for the Q-out-of-P fusion based detection technique is given by,

(16) $$P_{M,c}=\sum_{j=0}^{Q-1}\frac{P!}{j!(P-j)!}P_{D}^{j}{\left(1-P_{D}\right)}^{P-j}$$

(17) $$P_{FA,c}=1-\sum_{j=0}^{Q-1}\frac{P!}{j!(P-j)!}P_{FA}^{j}{\left(1-P_{FA}\right)}^{P-j}$$

Furthermore, two of the well-known logical operators, the OR function and the AND function, have also been used for the hard fusion decision. These two functions are special cases of the Q-out-of-P rule. The OR rule is implemented by letting *Q* = 1 and the AND rule is implemented by letting Q=P.

### 4.1. Q-Out-of-P Hard Fusion

In this paper, we have analyzed Q-out-of-P, AND, and OR cases of hard fusion. Here, we choose SSVD [15] as a local detector for exploring hard fusion schemes.

**Figure 2:**
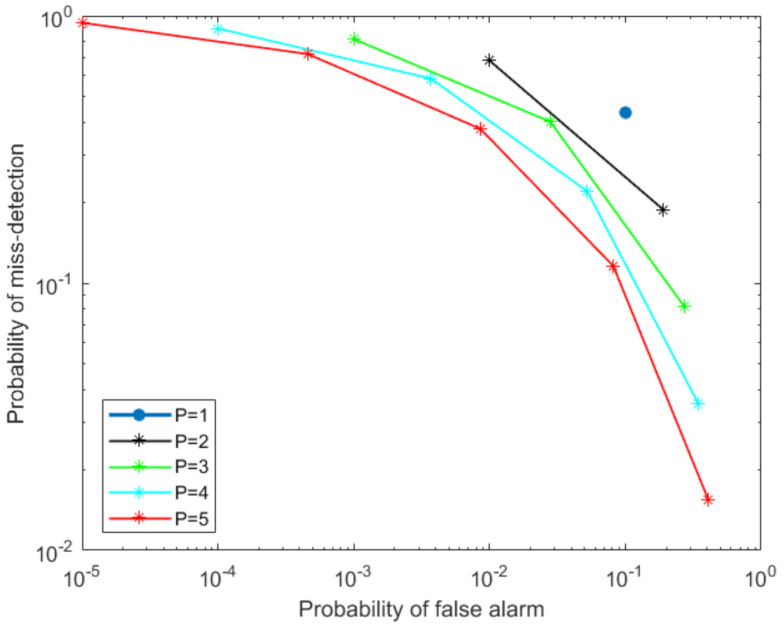
Q-out-of-P hard fusion strategy implemented using SSVD as a local detector.

We start by implementing Q-out-of-P-based fusion by keeping a constant probability of detection $P_{D}$ for all local SSVD detectors when the number of singular vectors is two, hence making the degree of freedom four. The complementary receiver operating characteristic curves for the Q-out-of-P fusion technique when the number of local detectors increases from one to five is depicted in Figure 2. The figure also illustrates the performance of the non-cooperative case with *P* = 1. As observed from Figure 2, the detection performance improves considerably with an increasing number of local detectors reporting to the fusion center. Moreover, we also observe how cooperative fusion improves the detection performance over the non-cooperative fusion case (shown with *P* = 1).

Next, we analyze hard fusion for ‘OR’ and ‘AND’ schemes. This scenario is considered for two different cases (1) when all the detectors have a variable probability of detection (2) when all the detectors have a constant probability of detection. Both cases are explained as follows.

#### 4.1.1. Probability of Detection Is Variable

Suppose if $P_{D}=Pr\left[u_{k}\left(i\right)=1\;|\;H_{1}\right],\;\forall \left(i,k\right)$ is the detection probability and $P_{D}=Pr\left[u_{k}\left(i\right)=1\;|\;H_{1}\right],\;\forall \left(i,k\right)$ is the false alarm probability of the local decisions made at the local detector, then the overall misdetection $P_{M,c}$ and the false alarm $P_{FA,c}$ probabilities for the OR and AND based hard data fusion strategies for target detection when we consider variable probability of detection for all local receivers are given by

OR fusion

(18) $$P_{M,c}=\prod_{p=1}^{P}\left(1-P_{Dp}\right)$$

(19) $$P_{FA,c}=1-{\left(1-P_{FA}\right)}^{P}$$

AND fusion

(20) $$P_{M,c}=1-\prod_{p=1}^{P}\left(P_{Dp}\right)$$

(21) $$P_{FA,c}={\left(P_{FA}\right)}^{P}$$

#### 4.1.2. Probability of Detection Is Constant

The second case we consider is when the probability of detection is the same for all the local receivers. In this case, miss detection and false alarm probabilities for the OR and AND-based data fusion strategies becomes $P_{M,c}={\left(1-P_{D}\right)}^{P}$ and $P_{M,c}=1-{\left(P_{D}\right)}^{P}$, respectively. As we have fixed the probability of false alarm for the local receivers, therefore, probability of false alarm at the fusion center represented by $P_{FA,c}$ remains the same for both cases.

**Figure 3:**
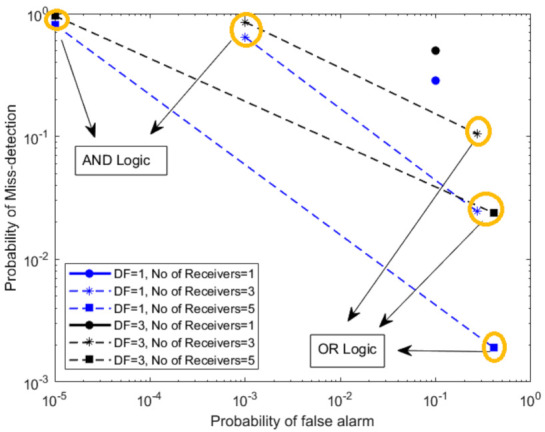
Hard fusion of SSVD detector with variable probability of detection for each receiver.

In Figure 3, we have evaluated hard fusion for the case of variable probability of detection for each receiver *P*. We have analyzed the results when the number of local detectors *P* are 1, 3, and 5. Here, we also monitor the performance of SSVD detector for various degrees of freedom (DF). We can observe the decreased performance as the degree of freedom is increased. The performance degrades due to the addition of noise vectors as the number of singular vectors increases by increasing the degree of freedom. Furthermore, the OR strategy displays better detection performance at the cost of the false alarm probability. In contrast, the AND strategy shows better false alarm performance at the expense of the detection probability. We can also observe the detector’s enhanced performance with an increasing number of local detectors *P*, regardless of the variable probability of detection.

In the next section, we will focus on applying soft fusion techniques for passive multistatic radars.

## 5. Soft Fusion Based Detection

As we know, in soft fusion, the local detector transmits the sensed data directly to the fusion center, and the fusion center combines the information to decide upon the presence of the target. Suppose the information at the $p^{th}$ detector is $T_{p}$, then the information received at the fusion center from P detectors can be written as,

(22) $$\tau =[T_{1},T_{2},......,T_{P}]$$

or

(23) $$T_{F}=\sum_{p=1}^{P}w_{p}T_{p}=w\tau^{T}$$

where $w=[w_{1},w_{2},......,w_{P}]$.

To analyze the soft fusion in a passive multistatic radar case, SVD has been used as a local detector. SVD is chosen among all detectors because, from the observation made in Section 6.1, the SVD detector outperforms all other practical detectors. Moreover, it is also a special case of SSVD detector when we consider only the largest singular vector.

### 5.1. Equal Gain Soft Fusion

Equal Gain is the most straight forward yet most commonly used blind soft fusion technique. In this technique, we consider fusing all the detectors by assigning equal weights $w_{p}=1$ to each local detector *p*. Hence, the fusion test statistics become

(24) $$T_{F}=\sum_{p=1}^{P}T_{p}$$

We calculate test statistics for each receiver for both the null hypothesis and the alternate hypothesis and pass it to the fusion center, where the fusion center sums them up with equal weights.

#### Threshold Calculation of Equal Gain Soft Fusion under Null Hypothesis

In this section, a closed-form expression for the threshold determining equal gain fusion decision has been calculated, specifically when an SVD detector, given by Equation (8) has been used as a local detector at each receiver. As we know, the distribution of the singular vectors is not known. However, in [14], Gaussian Perturbation Model has been used to express Singular Vector Distribution and later validated using simulation. Moreover, under the Null hypothesis, we know that the surveillance channel just consists of noise having Gaussian distribution. Therefore we can approximate the sample singular vector asymptotically as $v_{sp}∼\;\mathrm{CN}\left(0,\frac{I}{M}\right)$. Since the noise vectors in the surveillance and reference channels are statistically independent; for any given unit norm vector $v_{rp}$, we have $\left[{v_{rp}}^{H}v_{sp}\right]\;∼\;\mathrm{CN}\left(0,\frac{1}{M}\right)$. Therefore, under null hypothesis, equal gain soft fusion of SVD based local detector given by

(25) $$T_{F}=\sum_{p=1}^{P}{\left|v_{rp}^{H}v_{sp}\right|}^{2}$$

will follow, approximately, a chi-square distribution [44] with degree of freedom 2P, given by

(26) $$\frac{1}{2M}{\chi_{2P}}^{2}$$

here, p∈[1,...,P] and *P* are the number of local detectors in the system. ${\chi_{2P}}^{2}$ is Chi-Squared distribution with degree of freedom 2P. The degree of freedom depends on the number of local detectors fused. Let ζ be the detection threshold. Then,

(27) $$P\left(\frac{1}{2M}{\chi_{2P}}^{2}>\zeta \right)=P\left({\chi_{2P}}^{2}>2M\zeta \right)=P_{FA}$$

(28) $$P_{FA}=1-\frac{\gamma (\frac{2P}{2},\frac{2M\zeta }{2})}{\Gamma (\frac{2P}{2})}$$

here, we consider the fact that the CDF of Chi-Squared distribution is equivalent to

(29) $$F_{\chi_{2P}}\left(x=2M\zeta \right)=\frac{\gamma (\frac{2P}{2},\frac{2M\zeta }{2})}{\Gamma (\frac{2P}{2})}$$

where Γ and γ are the gamma function and lower incomplete gamma function, respectively. Thus, the detection threshold for any given probability of false alarm $P_{FA}$ can be shown by the closed-form expression calculated as

(30) $$\zeta =\frac{\gamma^{-1}\left(\left(1-P_{FA}.\Gamma \left(P\right)\right),P\right)}{M}$$

It is noteworthy that the detection threshold is independent of received signal power and SNR, which is a highly desirable feature of Radar detection.

**Figure 4:**
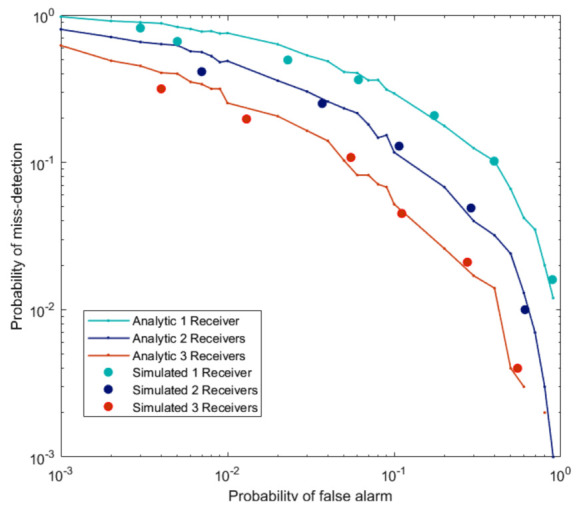
Validation of *P_FA_* defined in Equation (28) for Equal gain soft fusion test statistic (SVD as a local detector) for Surveillance SNR = −10 dB and Reference SNR = −10 dB.

In Figure 4, we compare the equal gain soft fusion method (SVD as a local detector) for a different number of receivers. We have used Equation (28) for the probability of false alarm, and the probability of detection is obtained using Monte-Carlo simulations. On the other hand, for simulated results, both the false alarm probability and the detection probability are calculated using Monte-Carlo simulations. Obtained results validate each other, hence proving our analysis for threshold calculation under null hypothesis accurate.

### 5.2. Singular Values Based Proposed Soft Fusion Techniques

In the analysis phase, we observed that the performance of the technique equal gain fusion could be improved upon, as this fusion method gives equal weights to each local detector. It does not exploit the fact that some local detectors may receive a better signal than others. As in practical cases, signal attenuation for each receiver is not constant, and hence the resulting signal-to-noise ratio in the surveillance channel may vary for each local receiver. If the received signal contains signal and noise, then the largest singular value primarily reflects the power concentration of the initially transmitted signal. Hence, we utilize the singular values embedded inside the received signal.

In particular, we propose two main fusion techniques based on singular values and four more designs that are precisely the variations of the first two proposed techniques.

#### 5.2.1. Proposed Technique 1

The combination of maximum singular value and trace obtained from the sample covariance matrix of the received signal matrix plays a vital role in various hypothesis testing problems, both in statistics and in signal processing. This combination can contribute to fusion decision by sensing the amount of signal in each local detector. Considering this, the weights defined in Equation (23) can be expressed as follow,

(31) $$w_{p}=\frac{max\;(S_{s_{\left(p\right)}})}{trace\;(S_{s_{\left(p\right)}})}$$

where $S_{s_{\left(p\right)}}$ is the singular value matrix obtained by taking SVD of received signal $Y_{s_{\left(p\right)}}$.

#### 5.2.2. Proposed Technique 2

Another significant result from random matrix theory is the condition number method. The condition number method represents the ratio of the largest and smallest eigenvalue. In our second proposed algorithm, inspired by this method, we use the ratio of the largest and smallest singular value obtained by the singular value decomposition of received data matrix. Hence, the weights $w_{p}$ become,

(32) $$w_{p}=\frac{max\;(S_{s_{\left(p\right)}})}{min\;(S_{s_{\left(p\right)}})}$$

#### 5.2.3. Proposed Technique 3

This technique is a variation of proposed technique 1. In this technique, we have applied the logarithmic function to the base 10 on proposed technique 1. Generally, the logarithm is a slowly increasing function, and it compresses large-scale data. The mathematical expression of this technique is given by

(33) $$w_{p}=\log\left[1+\frac{max\;(S_{s_{\left(p\right)}})}{trace\;(S_{s_{\left(p\right)}})}\right]$$

#### 5.2.4. Proposed Technique 4

The exponential function is one of the most critical functions in mathematics. It increases rapidly, so here in this technique, we have analyzed its effect when applied on singular values divided by its trace. Mathematically, we can express it as

(34) $$w_{p}=\exp\left[\frac{max\;(S_{s_{\left(p\right)}})}{trace\;(S_{s_{\left(p\right)}})}\right]$$

#### 5.2.5. Proposed Technique 5

In this fusion technique, instead of applying the exponential on a ratio, we apply it on the largest singular value present in the singular value matrix, given by

(35) $$w_{p}=\exp\left[max\left[S_{s_{\left(p\right)}}\right]\right]$$

#### 5.2.6. Proposed Technique 6

In this technique, we have applied the logarithmic function to the base ten on largest singular value present. The mathematical expression of the method is as follows:

(36) $$w_{p}=\log\left[1+max\left[S_{s_{\left(p\right)}}\right]\right]$$

## 6. Simulation and Performance Analysis

In this section, numerical simulation results are provided to verify the analysis done so far and evaluate the performance of the proposed techniques. MATLAB has been used as a software platform to execute simulations. In all the simulations, the values of standard parameters used are provided in Table 1.

**Figure 5:**
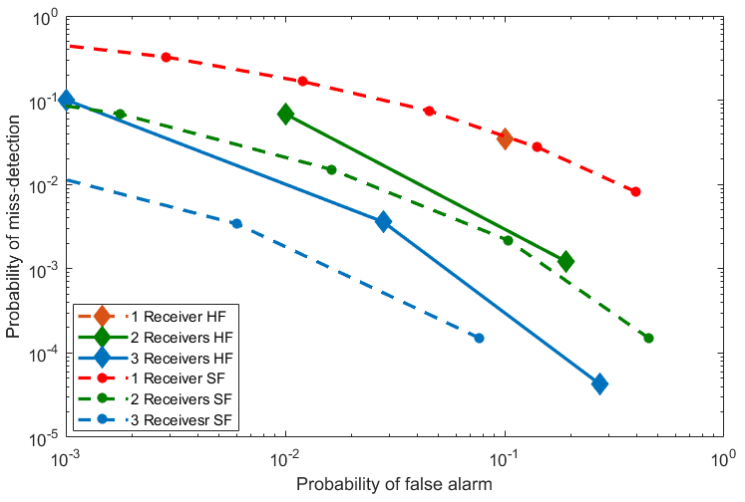
Comparison of soft fusion (SF) and hard fusion (HF).

In Figure 5, we have compared the two basic fusion methods, i.e., hard fusion and soft fusion. This figure illustrates the fusion techniques for the case of 1, 2, and 3 spatially separated local receivers, respectively. Here, we consider SVD detector as a local receiver. The surveillance SNR and the reference SNR are set to be −10 dB and −10 dB, respectively. Here, the hard fusion is plotted theoretically using Equations (18) and (19) where $P_{FA}$ is assumed to be 0.1. Soft fusion is simulated using the Monte-Carlo counting technique. We observe that as the number of local detectors increases from one, soft fusion starts outperforming hard fusion. Here, we have used equal gain combining as soft fusion technique and Q-out-of-P as hard fusion technique.

### 6.1. Hard Fusion

In hard fusion-based detection, the local detector transmits its binary decisions on the presence of a target in the environment to the fusion center. This process has no liberty to use any other detection technique except the mentioned Q-out-of-P, AND, and OR techniques. Therefore, the advancement that can be done is minimal. However, we can use an optimal local detector that can improve the whole system’s detection performance. For this purpose, we have exploited some recently developed radar detector and analyzed them in the framework of hard fusion.

The details of detectors we consider for performance analysis are given in Section 3.1.2. In particular, the response of local detectors test statistics for the hard fusion framework is analyzed. We use Q-out-of-P fusion strategy. Here, we use constant $P_{D}$ for each local receiver to analyze the system on a precisely similar platform. The values of other parameters considered are given in Table 1. For transmitted pulse *u*, any periodic waveform can be used. Here, we have considered simple unit norm sinusoidal wave.

**Figure 6:**
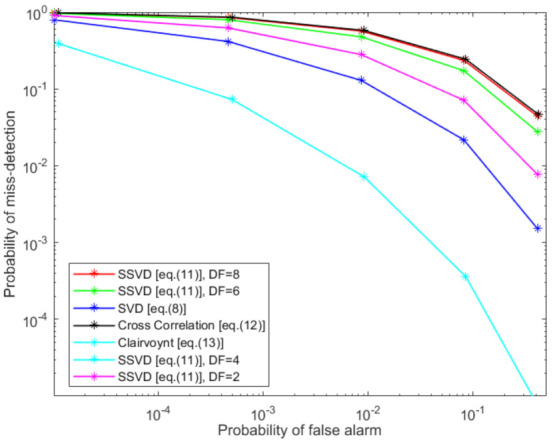
Hard fusion of detectors when number of receivers are 5.

The complementary receiver operating characteristics curves for the Q-out-of-P hard fusion technique for different local detectors is depicted in Figure 6. Here, we assume five receivers in the system. In Figure 6, we can clearly see that clairvoyant as the benchmark detector outperforms all other detectors. Moreover, the random matrix theory-based detectors, SVD and SSVD for different degrees of freedom (DF), perform better than conventional cross-correlation detector.

**Figure 7:**
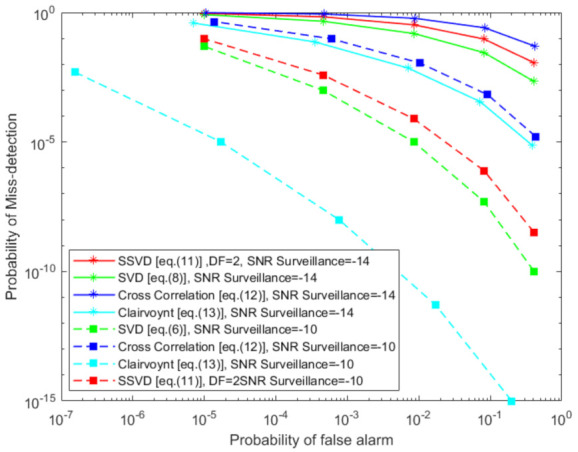
Hard fusion (Q-out-of-P) of detectors when the surveillance SNR increases for reference SNR = 0 dB.

We also analyze local detector’s performance in hard fusion framework when the SNR of the surveillance channel increases. In Figure 7, as the SNR of the surveillance channel increases from −14 dB to −10 dB. We observe that the performance of every detector increases by a considerable amount.

### 6.2. Soft Fusion

**Figure 8:**
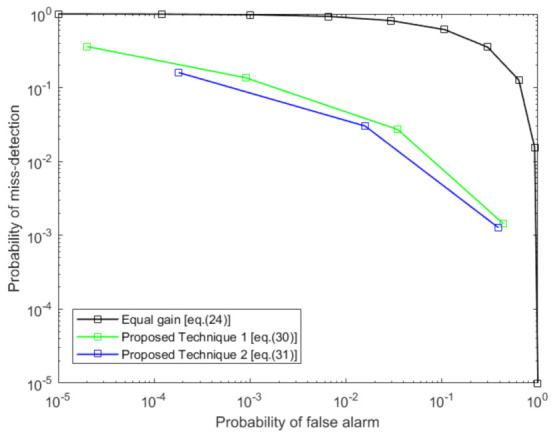
Comparison of proposed fusion techniques with equal gain fusion when surveillance SNR is −20, −18, −16, −14, −12 dB at each local detector *p*.

In Figure 8, we assume five local receivers. Each receiver has variable SNR in the surveillance channel taken as −20, −18, −16, −14, and −12 dB, respectively. In this figure, we have compared our two basic proposed techniques with equal gain fusion. Note that the SVD detector has been used for local detection, as we described earlier in the paper. We notice that the proposed techniques significantly outperform the equal gain fusion technique. As in our proposed technique, we ensure that the local detector with a high SNR value in the surveillance channel has more weight than the detector with low SNR in the surveillance channel, unlike the equal gain fusion technique. Moreover, we also observe that proposed technique 2 performs slightly better than proposed technique 1.

**Figure 9:**
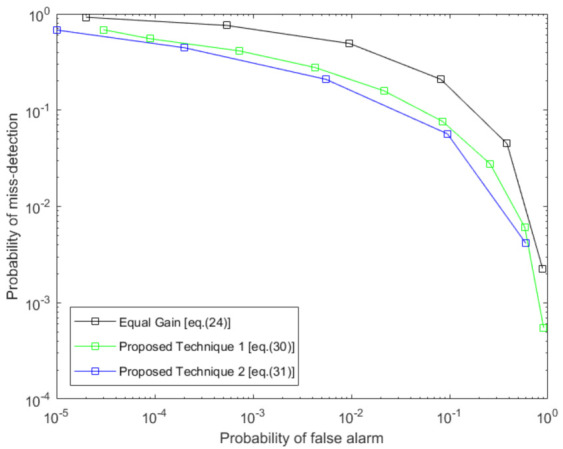
Comparison of proposed fusion techniques with equal gain fusion when surveillance SNR is −28, −24, −20, −16, and −12 dB at each local detector *p*.

In Figure 9, we present the ROC curves for even lower surveillance SNR values of our major proposed techniques and equal gain fusion. The SNR values in the surveillance channel are taken as −28, −24, −20, −16, and −12 dB, respectively. We observe that even with a very low SNR of the surveillance channel, our proposed techniques perform better than equal gain fusion, as our proposed techniques are more dependent on detectors receiving a less attenuated signal.

**Figure 10:**
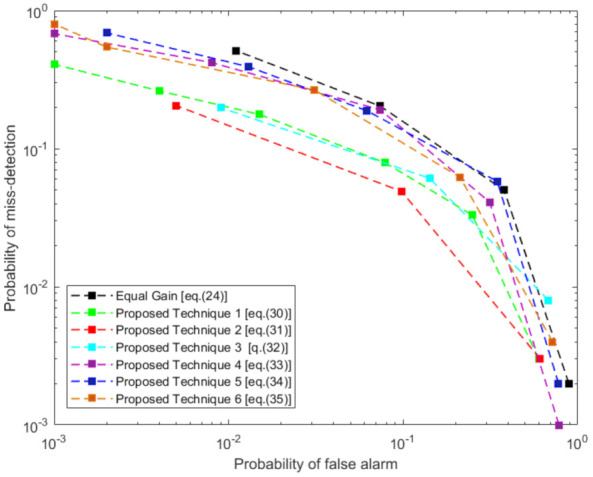
Comparison of proposed fusion techniques with equal gain fusion when surveillance SNR is −28, −24, −20, −16, and −12 dB at each local detector *p* and Reference SNR = −15 dB.

In Figure 10, we have analyzed the remaining four proposed techniques. Here, we can see that although our proposed technique 2 outperforms all other techniques yet proposed, technique 2, 3, 4, and 6 can outperform the existing equal gain fusion technique. Hence we can clearly state that the singular value-based fusion techniques can outperform the equal gain fusion technique for any given probability of false alarm. While our proposed fusion techniques may need more computation than the equal gain fusion, as it requires computing the SVD of two received data signals, but it is not an iterative procedure. Hence, with the growing advancements in computing technology, it can be accomplished in the practical world.

## 7. Conclusions

In this paper, we have done a detailed analysis of the application of hard and soft fusion techniques for multistatic passive radars. Hard fusion techniques were analyzed for different local detectors. Among all the detectors, the particular case of SSVD detector, also known as SVD detector, showed the best performance. Moreover, hard fusion techniques were also analyzed for the case when the probability Of detection is constant and when the probability of detection is not constant. Additionally, we have studied the performance of equal gain soft fusion while considering SVD as a local detector. We derived an approximate expression for the detection threshold under the null hypothesis. Furthermore, we compared existing soft and hard fusion techniques and determined that the soft fusion techniques perform better than hard fusion techniques. Lastly, based on our research, we proposed some novel singular value-based soft fusion techniques. We demonstrated that the proposed techniques could significantly outperform the classical equal gain fusion when used in passive multistatic radar systems.

In the future, we will expand our research specifically for the case of multiple targets. Moreover, our preliminary results demonstrate improvement in detection performance using novel soft fusion techniques, and we will develop the analysis for these techniques in our future work. 

## Figures and Tables

**Table 1:** Parameters used in Numerical Simulations.

Parameter Name	Values
Iterations	1000, 100,000
M	11
N	50
Transmitted wave	Sinusoidal
Surveillance SNR	−14 dB, −10 dB
Reference SNR	0 dB, −10 dB
